# Beneficial effects of lingonberry (*Vaccinium vitis-idaea* L.) supplementation on metabolic and inflammatory adverse effects induced by high-fat diet in a mouse model of obesity

**DOI:** 10.1371/journal.pone.0232605

**Published:** 2020-05-07

**Authors:** Riitta Ryyti, Mari Hämäläinen, Rainer Peltola, Eeva Moilanen

**Affiliations:** 1 The Immunopharmacology Research Group, Faculty of Medicine and Health Technology, Tampere University and Tampere University Hospital, Tampere, Finland; 2 Natural Resources Institute Finland, Bioeconomy and environment, Rovaniemi, Finland; University of Cambridge, UNITED KINGDOM

## Abstract

Obesity is a constantly increasing health problem worldwide. It is associated with a systemic low-grade inflammation, which contributes to the development of metabolic disorders and comorbidities such as type 2 diabetes. Diet has an important role in the prevention of obesity and its adverse health effects; as a part of healthy diet, polyphenol-rich berries, such as lingonberry (*Vaccinium vitis-idaea* L.) have been proposed to have health-promoting effects. In the present study, we investigated the effects of lingonberry supplementation on high-fat diet induced metabolic and inflammatory changes in a mouse model of obesity. Thirty male C57BL/6N mice were divided into three groups (n = 10/group) to receive low-fat (LF), high-fat (HF) and lingonberry-supplemented high-fat (HF+LGB) diet for six weeks. Low-fat and high-fat diet contained 10% and 46% of energy from fat, respectively. Lingonberry supplementation prevented the high-fat diet induced adverse changes in blood cholesterol and glucose levels and had a moderate effect on the weight and visceral fat gain, which were 26% and 25% lower, respectively, in the lingonberry group than in the high-fat diet control group. Interestingly, lingonberry supplementation also restrained the high-fat diet induced increases in the circulating levels of the proinflammatory adipocytokine leptin (by 36%) and the inflammatory acute phase reactant serum amyloid A (SAA; by 85%). Similar beneficial effects were discovered in the hepatic expression of the inflammatory factors CXCL-14, S100A10 and SAA by lingonberry supplementation. In conclusion, the present results indicate that lingonberry supplementation significantly prevents high-fat diet induced metabolic and inflammatory changes in a murine model of obesity. The results encourage evaluation of lingonberries as a part of healthy diet against obesity and its comorbidities.

## 1. Introduction

The prevalence of obesity, metabolic syndrome and type 2 diabetes has increased rapidly worldwide during the last decades. Reasons can be found in changing lifestyles, which lead to reduced physical activity and obesogenic diet. It seems that obesity is continuing to be an increasing global health burden; according to the WHO statistics, 13% of adults aged 18 and over (corresponding to over 650 million people) were obese (body mass index BMI > 30 kg/m^2^) and 39% overweight in 2016 [1]. Obesity is a complex chronic disorder with a multifactorial etiology, involving genetics, hormones, diet and environment [2], and it has a major impact on various metabolic and (patho)physiological functions in the human body. Obesity is a major risk factor and underlying condition in the progression of many metabolic disorders, particularly type 2 diabetes, cardiovascular diseases and cancer, through its effects on the development of, for instance hypertension, insulin resistance, nonalcoholic fatty liver disease and inflammation [3,4].

Adipose tissue is an active tissue regulating various physiological and pathological processes, including immunity and inflammation. It is therefore no longer considered only as a passive energy storage. Adipose tissue produces and releases several hormone-like factors called adipokines, and many of them have pro- or anti-inflammatory properties [2,5]. In obese adipose tissue, immune cells secreting pro-inflammatory substances increase in number while those producing anti-inflammatory substances have been shown to decrease. This imbalance is responsible for the obesity induced low-grade inflammation and insulin resistance in the body [3].

Diet has an important role in the prevention and treatment of obesity, type 2 diabetes and other obesity-related diseases. Diets containing plenty of polyphenol-rich vegetables have been shown to lower the risk of obesity-related comorbidities [6–8]. Berries are specifically rich in various polyphenols [9,10]. Diets containing berries are associated with lowered risk of type 2 diabetes, probably due to the flavonoids [11], anthocyanidins [8,12,13] or other polyphenols [14] present in berries. Several intervention studies have shown beneficial effects of berries also on inflammation and cardiovascular diseases [15].

Lingonberry (*Vaccinium vitis-idaea* L.) has been reported to have promising health-beneficial effects and anti-inflammatory properties in experimental models [16–19]. Lingonberries / lingonberry extracts were found to exhibit antidiabetic potential in various *in vitro* tests [20] and beneficial metabolic effects in mice exposed to high-fat diet [21–25]. Lingonberries are rich in dietary polyphenols with high antioxidant activities [16,26–28] and contain also essential omega-3 fatty acids [29] and plant sterols [30,31], which may contribute to their health-promoting effects. Lingonberries are commonly consumed in the Nordic countries and commercially available in many different forms. They are also the most generally collected and commercially utilized wild berries in Finland [32].

In the present study, we aimed to investigate the effects of lingonberry supplementation on metabolic and inflammatory changes in high-fat diet induced experimental obesity in mice to extend the current understanding on the health benefits of lingonberries. As the test material, we used commercially available air-dried lingonberry powder made from Finnish lingonberries.

## 2. Materials and methods

### Animals and study design

Male C57BL/6N mice (Scanbur Research A/S, Karlslunde, Denmark), 8 weeks of age and 24.3±0.2g of weight at the beginning of the experiment, were divided into three groups of 10 mice, and housed two mice per cage in the animal facility of the Tampere University under standard conditions (12/12h light/dark cycle, 22±1 °C temperature, and 50–60% humidity) with food and water provided *ad libitum*.

The mice were fed with normal low-fat diet (LF, 10 kcal% fat), with high-fat diet (HF, 46 kcal% fat) or with high-fat diet supplemented with air-dried lingonberry (*Vaccinium vitis-idaea* L.) powder (HF + LGB, 20% w/w) for 6 weeks. Both high-fat diets contained 46% of energy from fat and 36% from carbohydrate, while the low-fat diet had 10% of energy from fat and 72% from carbohydrate (Table 1). Otherwise the custom-made pelleted diets (Research Diets, Inc, New Brunswick, NJ, USA) were matched for protein (18% of energy from protein), fiber, vitamin and trace element contents considering the composition of the lingonberry powder. Air-dried lingonberry powder (100 g powder corresponds to ca 900 g fresh berries) was produced from Finnish lingonberries by Kiantama Ltd (Suomussalmi, Finland).

**Table 1:** Composition of the experimental diets.

	LF	HF	HF+LGB
Calculated energy (kcal)			
Protein	716	716	716
Carbohydrate	2840	1422	1422
Starch	2110	691	691
Sugar	730	731	731
Fat	405	1823	1823
Total energy	3961	3961	3961
Calculated energy per gram diet (kcal/g)	3.60	4.39	4.30
Calculated Energy (kcal%)			
Protein	18	18	18
Carbohydrate	72	36	36
Fat	10	46	46
Fiber (g%)	9	10	10
Lingonberry powder (g)	0	0	184*
Ingredients (g)			
			*(+ from LGB powder)*
Casein	200	200	194 *(+ 6)* total: 200
L-Cystine	3	3	3
Corn Starch	452	73	31 *(+ 42)* total: 73
Maltodextrin 10	75	100	100
Sucrose	173	173	103 *(+ 70)* total: 173
Cellulose	94	94	50 *(+ 44)* total: 94
Soybean Oil	25	25	24 *(+ 1)* total: 25
Lard	20	178	178
Mineral Mix S10026	10	10	10
DiCalcium Phosphate	13	13	13
Calcium Carbonate	6	6	6
Potassium Citrate	17	17	17
Vitamin Mix V10001	10	10	10
Choline Bitartrate	2	2	2

LF = low-fat diet, HF = high-fat diet, HF+LGB = lingonberry-supplemented high-fat diet

*Nutrient content/100 g lingonberry powder: fat 0.8 g, carbohydrates 61 g (of which sugars 38 g), fiber 24 g, protein 3 g

Body weight of the mice and food consumption was monitored weekly. At the end of the study, 6h-fasted mice were anesthetized with isoflurane (Oriola Corp., Espoo, Finland), blood glucose was measured and blood was collected by cardiac puncture. Tissue samples were collected for further analyses. The study was approved by the National Animal Experimental Board (permission number ESAVI- 984/04.10.07/2018) and the experiments were carried out in accordance with the EU legislation for the protection of animals used for scientific purposes (Directive 2010/63/EU).

### Blood samples and analyses

Six-hour fasting (morning fast) blood glucose levels in mice were measured from the tip of the tail with Contour Next One (Oy Diabet Ab, Lemu, Finland). Blood collected by cardiac puncture was centrifuged for 15 minutes at 1500 x g after 30 minutes incubation at room temperature, and obtained serum was immediately storaged at -80 °C. Serum triglyceride and total cholesterol levels, and alanine aminotransferase (ALT) activity were measured by fluorometric assays (Abcam, Cambridge, UK). Enzyme-linked immunoassays were used to measure the concentrations of leptin, resistin and adiponectin (R&D Systems Europe Ltd., Abingdon, UK), insulin (Mercodia Ltd., Uppsala, Sweden) and serum amyloid A (Tridelta Development Ltd., Maynooth, Ireland) in serum samples. Detection limits were 7.8 pg/mL for leptin and resistin, 15.6 pg/mL for adiponectin, 33 pmol/L for insulin and 16 ng/mL for SAA.

### RNA extraction and qRT-PCR

Total RNA was extracted from liver using RNeasy Mini Kit (Qiagen Inc., Hilden, Germany). Briefly, samples stored immediately after collection in RNA Later^®^ (Ambion, Thermo Fisher Scientific, Waltham, MA, USA) were weighed and maximum of 30 mg of tissue was cut into smaller pieces and homogenized with Qiashredder (Qiagen). RNA was extracted with RNeasy Mini Kit with on-column DNase digestion. RNA was transcribed to cDNA by using Maxima First Strand cDNA Synthesis Kit (Thermo Fisher Scientific) in 10 ul reaction volume and diluted 1:5 with RNase-free water. Quantitative PCR was performed using TaqMan Universal Master Mix and ABI Prism 7500 sequence detection system (Applied Biosystems, Foster City, CA, USA). The PCR cycling parameters were incubation at 50 °C for 2 minutes, incubation at 95 °C for 10 minutes, and thereafter 40 cycles of denaturation at 95 °C for 15 s and annealing and extension at 60 °C for 1 minute. Primers and probe for the housekeeping gene glyceraldehyde 3-phosphate dehydrogenase GAPDH) were GCATGGCCTTCCGTGTTC (forward, 300 nM), GATGTCATCATACTTGGCAGGTTT (reverse, 300nM) and TCGTGGATCTGACGTGCCGCC (probe, 150 nM); and TCGGAGGCTTAATTACACATGTTC (forward, 900 nM), CAAGTGCATCATCGTTGTTCATAC (reverse, 300 nM) and CAGAATTGCCATTGCACAACTCTTTTCTCA (probe, 200 nM) for interleukin 6 (IL-6). The sequences and concentrations were optimized according to the manufacturer’s guidelines in TaqMan Universal PCR Master Mix Protocol part number 4304449 revision C (Applied Biosystems). TaqMan Gene Expression assays for interleukin 1β (IL-1β, Mm00434228_m1), monocyte chemoattractant protein 1 (MCP-1, Mm00441242_m1), tumor necrosis alpha (TNF-α, Mm00443260_g1), glucose transporter 2 (GLUT2, Mm00446229_m1), serum amyloid A2 (SAA2, Mm04208126_mH), C-X-C motif chemokine ligand 14 (CXCL-14, Mm00444699_m1), S100 calcium-binding protein A10 (S100A10, Mm00501458_g1), and insulin receptor (Insr, Mm01211875_m1) were used (Thermo Fisher Scientific) and expression levels were calculated using the 2(−ΔΔCT) method. When calculating results, all of the mRNA expression levels were first normalized against GAPDH mRNA levels.

### Statistics

Results are expressed as mean + standard error of mean (SEM). One-way and two-way ANOVA with Bonferroni’s post-test, and the analysis of covariance were performed using GraphPad InStat version 3.10 and GraphPad Prism 8 (GraphPad Software, San Diego, USA), and IBM SPSS Statistics version 25.0 (IBM Corporation, Armonk, NY, USA). Asterisks *, **, and *** indicate p values smaller than 0.05, 0.01 and 0.001, respectively.

## 3. Results

### 3.1. Weight gain

**Fig 1:**
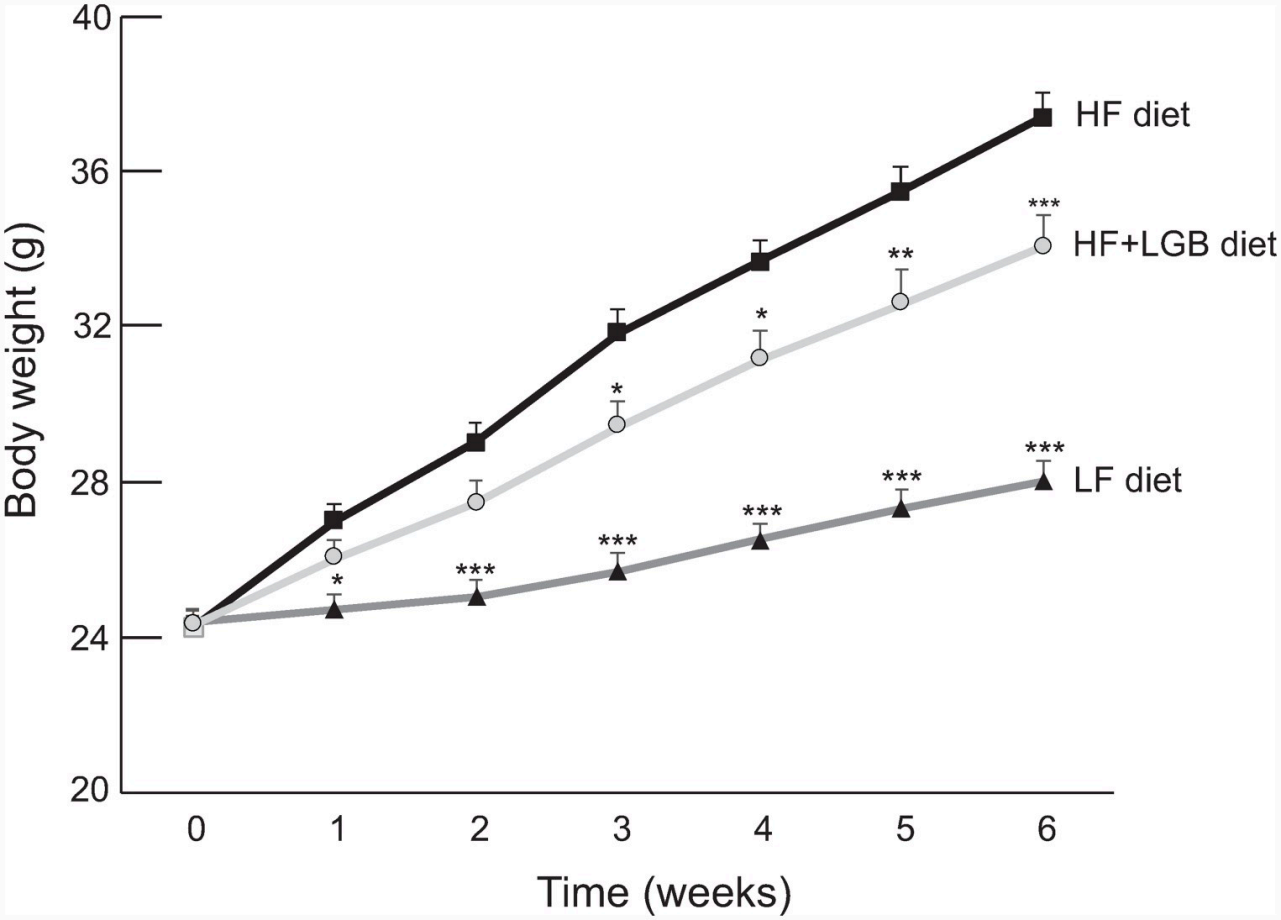
Body weight gain of the mice during the study. Animals received low-fat diet (LF diet, 10% of energy from fat, dark grey line), high-fat diet (HF diet, 46% of energy from fat, black line) or high-fat diet supplemented with lingonberry (HF + LGB diet, light grey line). Weight was measured once a week. The results are expressed as grams (g). Values represent mean + SEM, *n* = 10 mice per group. Repeated measures two-way ANOVA with Bonferroni post-test was used in the statistical analysis. Mean values significantly different from the high-fat group (HF diet) are marked with *p < 0.05, **p < 0.01 and ***p < 0.001.

The weight of mice in the high-fat (HF) group increased considerably during the study as compared to the mice in the low-fat (LF) control group. Interestingly, lingonberry supplementation prevented significantly the high-fat diet induced weight gain (p < 0.001 between HF and HF+LGB groups). After 6 weeks, the average weight of the low-fat group was 28.0±0.4 g, 37.4±0.6 g in the high-fat group and 34.1±0.6 g in the lingonberry supplemented high-fat group, respectively (p < 0.001 between groups). The development of weight of the mice in the test groups is presented in the Fig 1.

**Fig 2:**
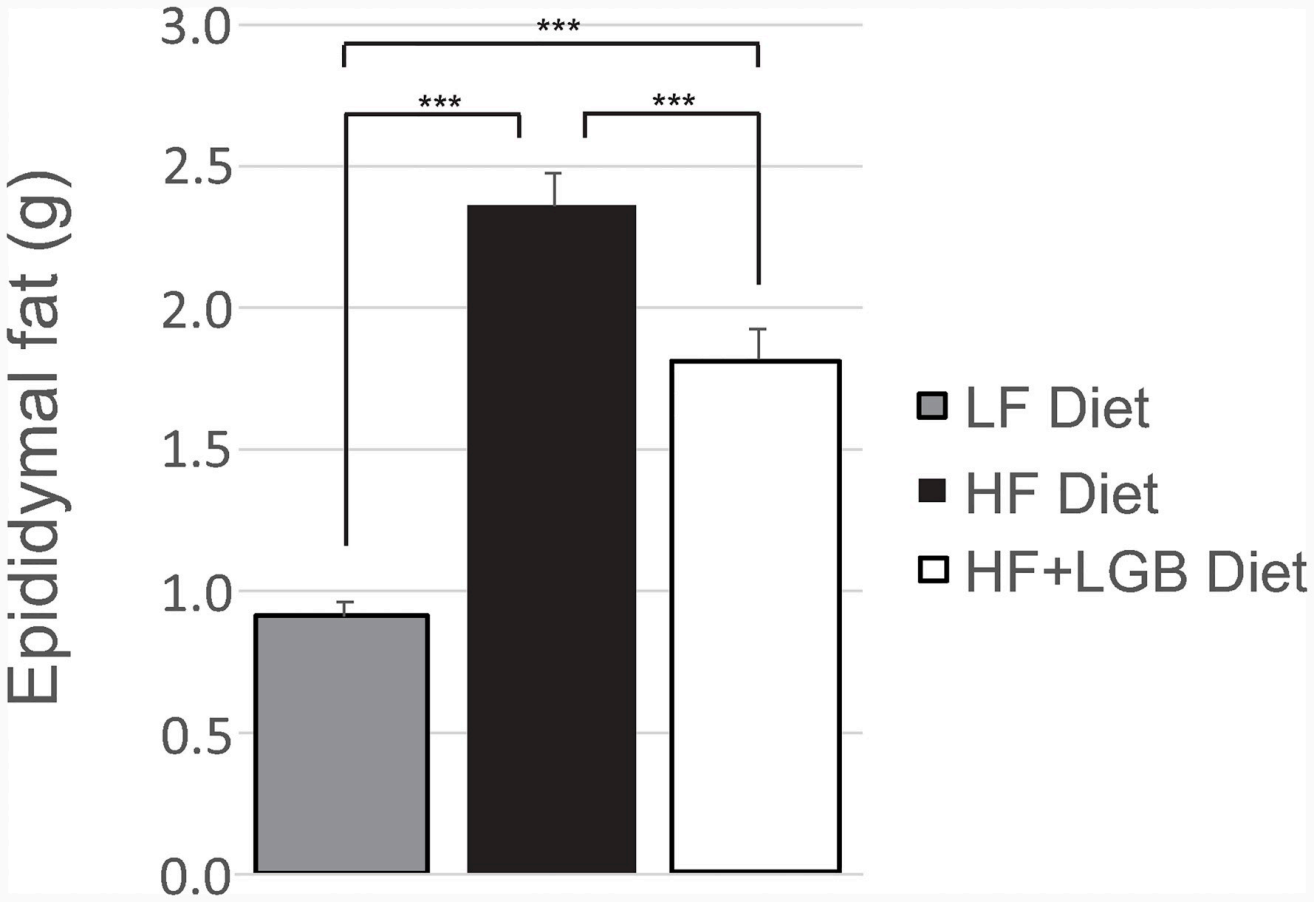
The amount of epididymal fat of the mice at the end of the study. Animals received low-fat diet (LF diet, 10% energy from fat, grey column), high-fat diet (HF diet, 46% energy from fat, black column) or high-fat diet supplemented with lingonberry (HF + LGB diet, white column). The amount of epididymal fat was measured at the end of the study. The results are expressed as grams (g). Values represent mean + SEM, *n* = 10 mice per group. One-way ANOVA with Bonferroni post-test was used in the statistical analysis. Differences between the groups are marked with ***p < 0.001.

The amount of epididymal fat increased in the high-fat diet group, when compared to the low-fat control group (p < 0.001). Lingonberry supplementation prevented significantly the accumulation of epididymal fat when compared to the high-fat control group (p < 0.001). At the end of the study, the amount of epididymal fat was 2.4±0.1 g in the high-fat group and 1.8±0.1 g in the lingonberry supplemented high-fat group. Both high-fat groups had significantly (p < 0.001) higher amount of epididymal fat than the low-fat group (0.9±0.0 g; Fig 2). When the ratio of the epididymal fat to the whole-body mass was calculated, it was lower in the HF+LGB group than in the HF group (p < 0.01) suggesting that lingonberry supplementation prevents particularly the accumulation of the metabolically highly detrimental visceral adipose tissue. That was also supported by the fact that in the analysis of covariance when the body weight was set as a covariate, the epididymal fat mass was lower in the LF and the HF+LGB groups than in the HF group (p < 0.01).

**Fig 3:**
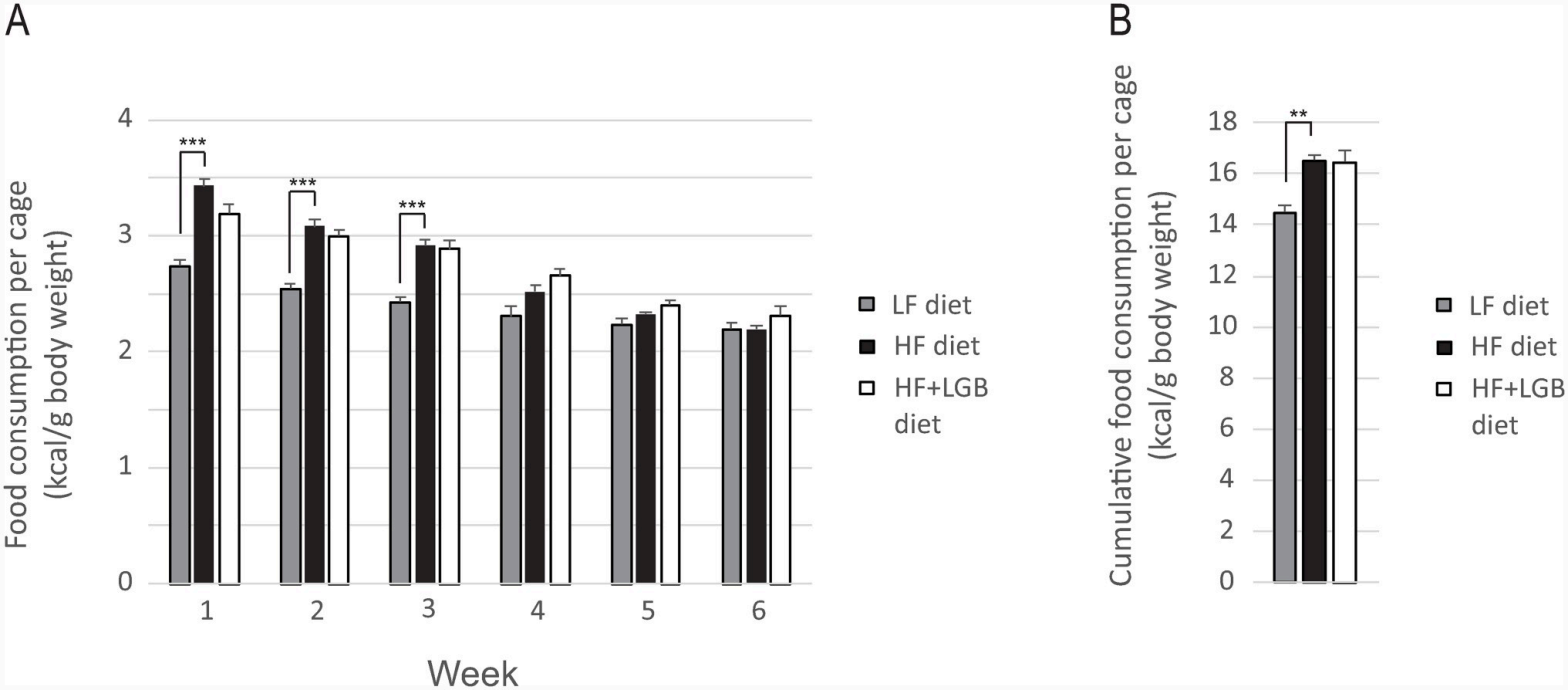
Food consumption during the study. Fig 3A shows the weekly and Fig 3B the cumulative food consumption during the six weeks’ study. Animals received low-fat diet (LF diet, 10% energy from fat, grey columns), high-fat diet (HF diet, 46% energy from fat, black columns) or high-fat diet supplemented with lingonberry (HF + LGB diet, white columns). Food consumption was measured once a week. The results are expressed as kcal/body weight in grams. Values represent mean + SEM, *n* = 10 mice per group; as the mice were housed two mice per cage, *n* = 5 was used in the statistical calculations. Repeated measures two-way ANOVA (Fig 3A) and one-way ANOVA (Fig 3B) with Bonferroni post-test was used in the statistical analysis. Differences between the groups are marked with **p<0.01 and ***p < 0.001.

Food consumption (kcal/g body weight) was measured weekly and it did not differ between the high-fat and the lingonberry supplemented high-fat diet groups although energy intake in the low-fat diet group was lower, particularly during the first half of the study (Fig 3A). We also calculated the cumulative food consumption (kcal/g body weight) during the six weeks’ study: no difference was found between the high-fat and the lingonberry supplemented high-fat groups while the value in the low-fat group was lower (p < 0.01; Fig 3B). This result was reproduced in repeated measures analysis of covariance with body weight as a covariate.

### 3.2. Glucose and insulin

**Fig 4:**
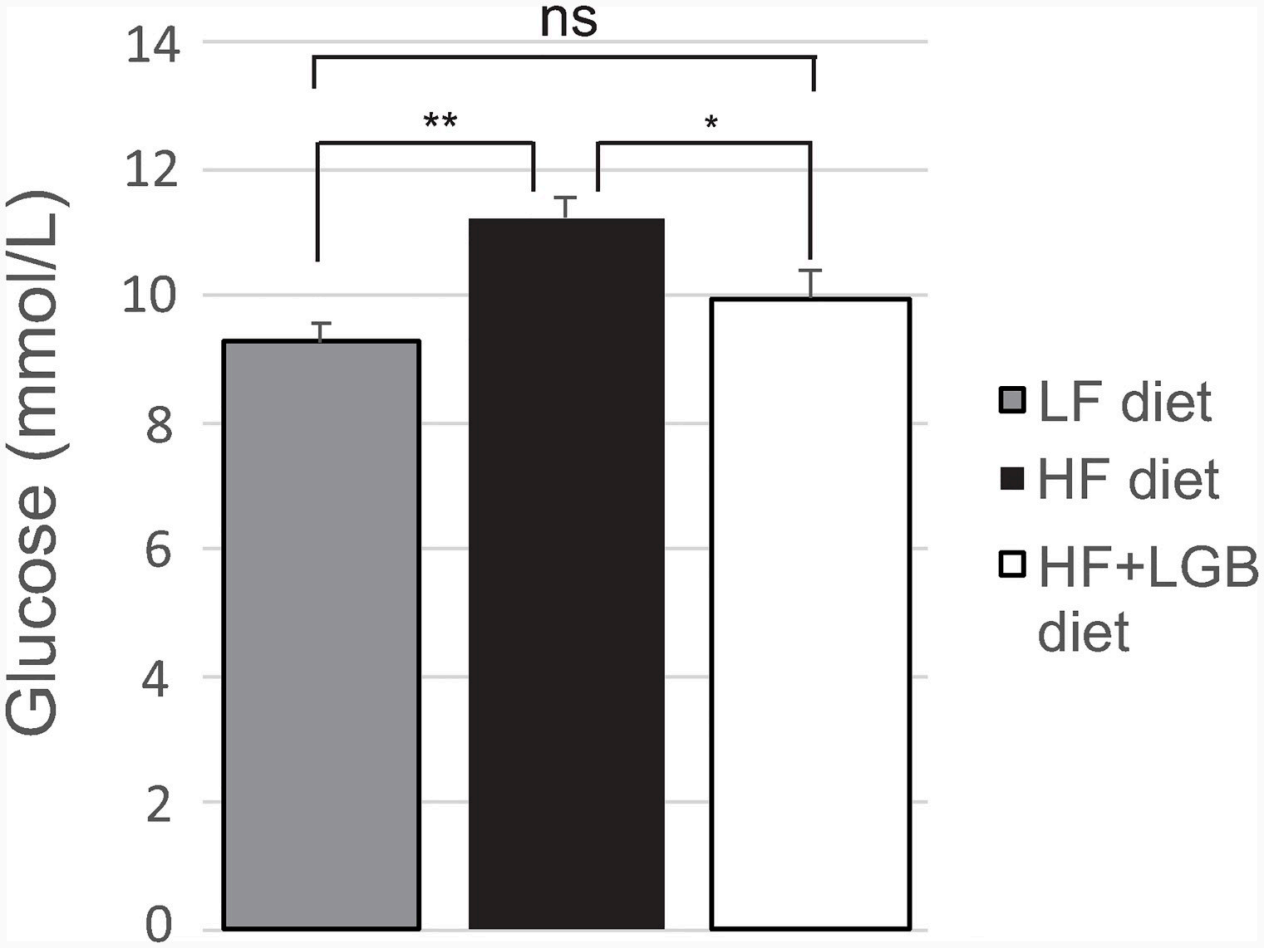
The fasting blood glucose levels of mice at the end of the study. Animals received low-fat diet (LF diet, 10% energy from fat, grey column), high-fat diet (HF diet, 46% energy from fat, black column) or high-fat diet supplemented with lingonberry (HF + LGB diet, white column). At the end of the study, blood samples for glucose measurements were collected after 6 h fasting. The results are expressed as mmol/L. Values represent mean + SEM, *n* = 10 mice per group. One-way ANOVA with Bonferroni post-test was used in the statistical analysis. Differences between the groups are marked with *p < 0.05, **p < 0.01 and ns = not significant.

Fasting blood glucose level at the end of the study was increased in the high-fat diet group, as compared to the low-fat control group (p < 0.01). Interestingly, in the lingonberry supplemented high-fat diet group, the glucose level (10.0±0.5 mmol/L) was lower than that in the high-fat control group (11.2±0.4 mmol/L; p < 0.05), and there was no statistically significant difference between the low-fat and the lingonberry supplemented high-fat groups (Fig 4).

**Fig 5:**
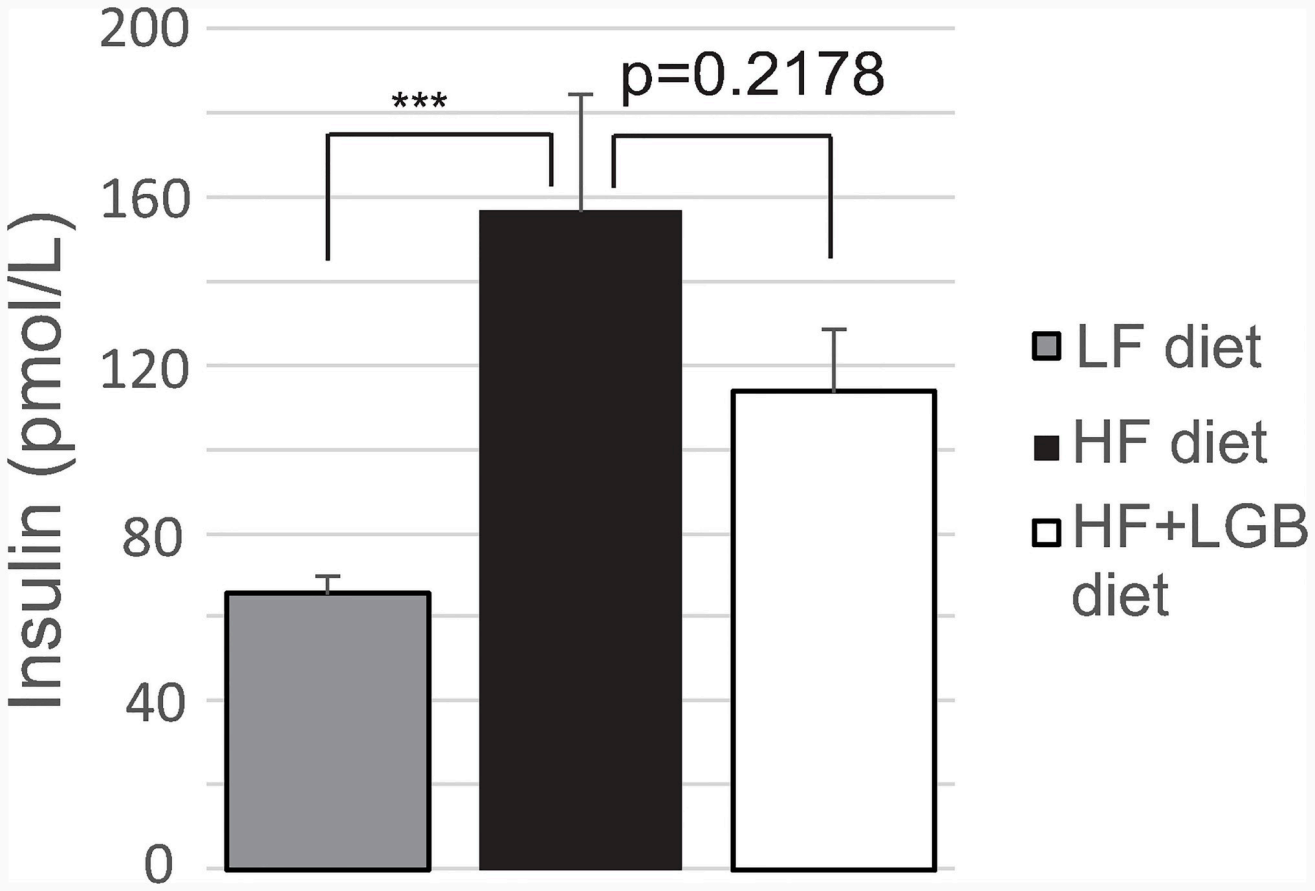
The insulin levels of mice at the end of the study. Animals received low-fat diet (LF diet, 10% energy from fat, grey column), high-fat diet (HF diet, 46% energy from fat, black column) or high-fat diet supplemented with lingonberry (HF + LGB diet, white column). At the end of the study, blood samples were collected after 6 h fasting and serum insulin concentrations were analyzed with ELISA. The results are expressed as pmol/L. Values represent mean + SEM, *n* = 10 mice per group. One-way ANOVA with Bonferroni post-test was used in the statistical analysis. Differences between the groups are marked with *** p < 0.001.

As expected, fasting insulin level was significantly higher in the high-fat group (156.5 pmol/L) as compared to the low-fat group (65.5 pmol/L; p < 0.001). In the lingonberry supplemented high-fat group, the insulin level (113.7 pmol/L) was lower than in the high-fat group, although the effect did not reach statistical significance (Fig 5).

### 3.3. Cholesterol and triglycerides

**Fig 6:**
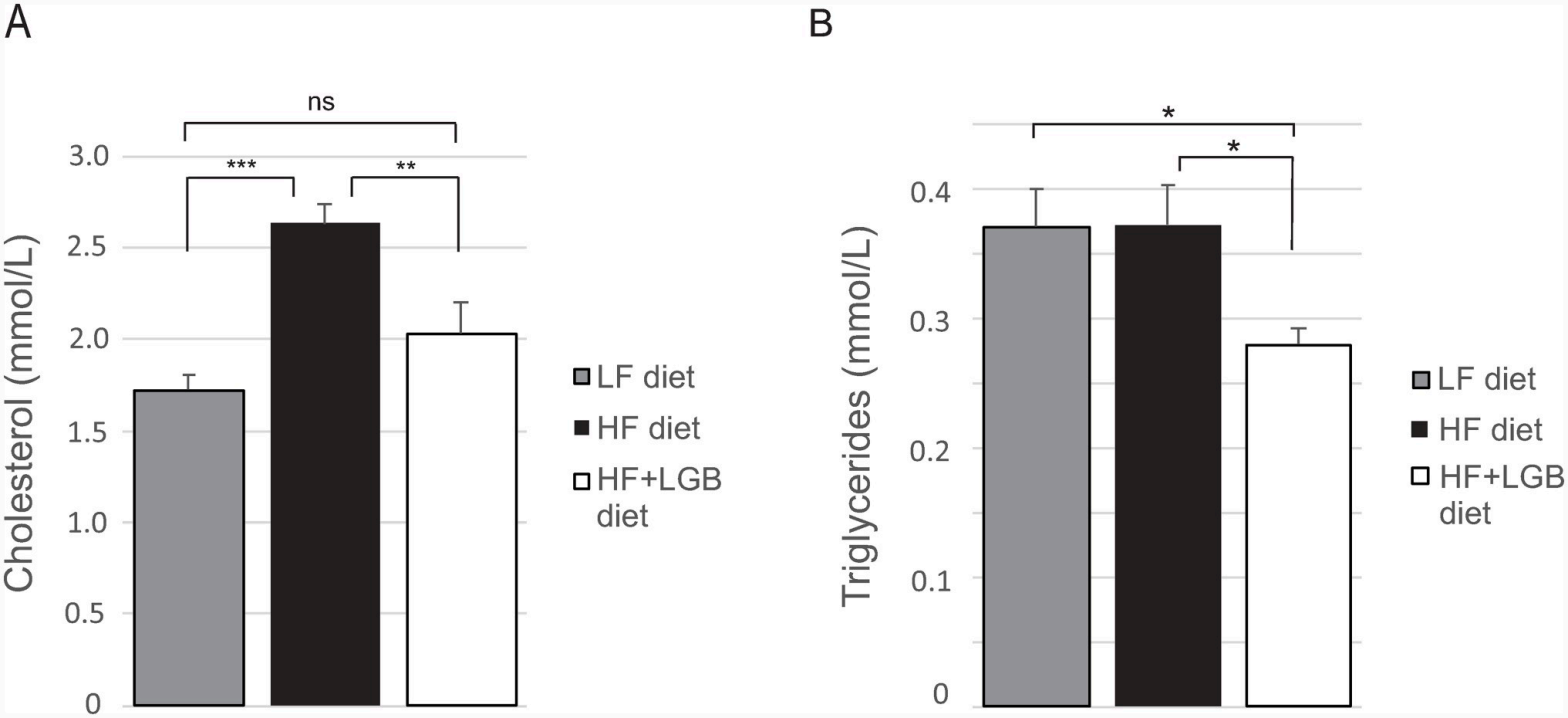
Cholesterol (A) and triglyceride (B) levels of mice at the end of the study. Animals received low-fat diet (LF diet, 10% energy from fat, grey columns), high-fat diet (HF diet, 46% energy from fat, black columns) or high-fat diet supplemented with lingonberry (HF + LGB diet, white columns). At the end of the study, blood samples were collected after 6 h fasting and serum cholesterol and triglyceride concentrations were analyzed with fluorometric assays. The values are presented as mmol/L. Values represent mean + SEM, *n* = 10 mice per group. One-way ANOVA with Bonferroni post-test was used in the statistical analysis. Differences between the groups are marked with *p < 0.05, **p < 0.01, ***p < 0.001 and ns = not significant.

Cholesterol level was significantly increased in the high-fat group when compared to the low-fat group (p < 0.001) being 2.6±0.1 mmol/L in the high-fat group and 1.7±0.1 mmol/L in the low-fat group at the end of the study. Importantly, the cholesterol level in the lingonberry supplemented high-fat group (2.0±0.2 mmol/L) was significantly lower when compared to the high-fat group (p < 0.01). There was no statistically significant difference in the cholesterol levels between the low-fat and the lingonberry supplemented high-fat groups indicating that lingonberry supplementation prevented the high-fat diet induced increase in cholesterol levels (Fig 6A).

There were no differences in the triglyceride levels between the low-fat and high-fat groups whereas the triglyceride level in the lingonberry supplemented high-fat group was lower than that in the two other groups (p < 0.05; Fig 6B).

### 3.4. Adipokines and inflammatory factors

As expected, leptin levels were significantly higher in the high-fat group as compared to the low-fat group (p < 0.001). Leptin levels were lower in the lingonberry supplemented high-fat group (27.1±3.5 ng/mL) than in the high-fat control group (39.7±2.8 ng/mL; p < 0.01, Table 2). Interestingly, the statistically significant difference was also observed in weight-related values (1.1±0.1 ng/mL/body weight (g) in HF group vs. 0.8±0.1 ng/mL/body weight (g) in HF+LGB group, p < 0.01). In adiponectin, there was a trend towards decreased levels in the high-fat group when compared to low-fat control group (p = 0.277). Adiponectin level was maintained at normal levels with lingonberry supplementation. There were no significant differences in the resistin levels between the groups (Table 2).

**Table 2:** The circulating adipokine and serum amyloid A (SAA) levels, and alanine aminotransferase (ALT) activity of the mice at the end of the study.

	Low-fat (LF)	High-fat (HF)	High-fat + lingonberry (HF+LGB)	p-value between LF and HF	p-value between HF and HF+LGB
Adiponectin (μg/mL)	6.9 ± 0.2	6.4 ± 0.2	7.1 ± 0.3	0.277	0.100
Leptin (ng/mL)	4.6 ± 0.6	39.7 ± 2.8	27.1 ± 3.5	< 0.001	< 0.01
Resistin (ng/mL)	17.8 ± 1.4	16.8 ± 0.8	17.6 ± 0.9	> 0.999	> 0.999
SAA (μg/mL)	6.6 ± 0.6	11.7 ± 0.7	7.4 ± 0.4	< 0.001	< 0.001
ALT (U/L)	8.1 ± 0.6	14.1 ± 0.8	7.2 ± 0.2	< 0.001	< 0.001

Adiponectin, leptin, resistin and SAA concentrations were measured by ELISA, and ALT activity by fluorometric assay. The results are presented as mean ± SEM; *n* = 10 mice per group. One-way ANOVA with Bonferroni post-test was used in the statistical analysis.

The levels of the inflammatory acute phase reactant serum amyloid A (SAA) were increased (p < 0.001) in the high-fat diet group (11.7±0.7 μg/mL) as compared to the low-fat diet group (6.6±0.6 μg/mL). Importantly, the SAA levels in the lingonberry supplemented high-fat group (7.4±0.4 μg/mL) were significantly lower (p < 0.001) than those in the high-fat control group. Similarly, alanine aminotransferase activity was increased in the high-fat diet group as compared to the low-fat diet group (p<0.001) which was prevented by the lingonberry supplementation (p < 0.001) (Table 2).

**Fig 7:**
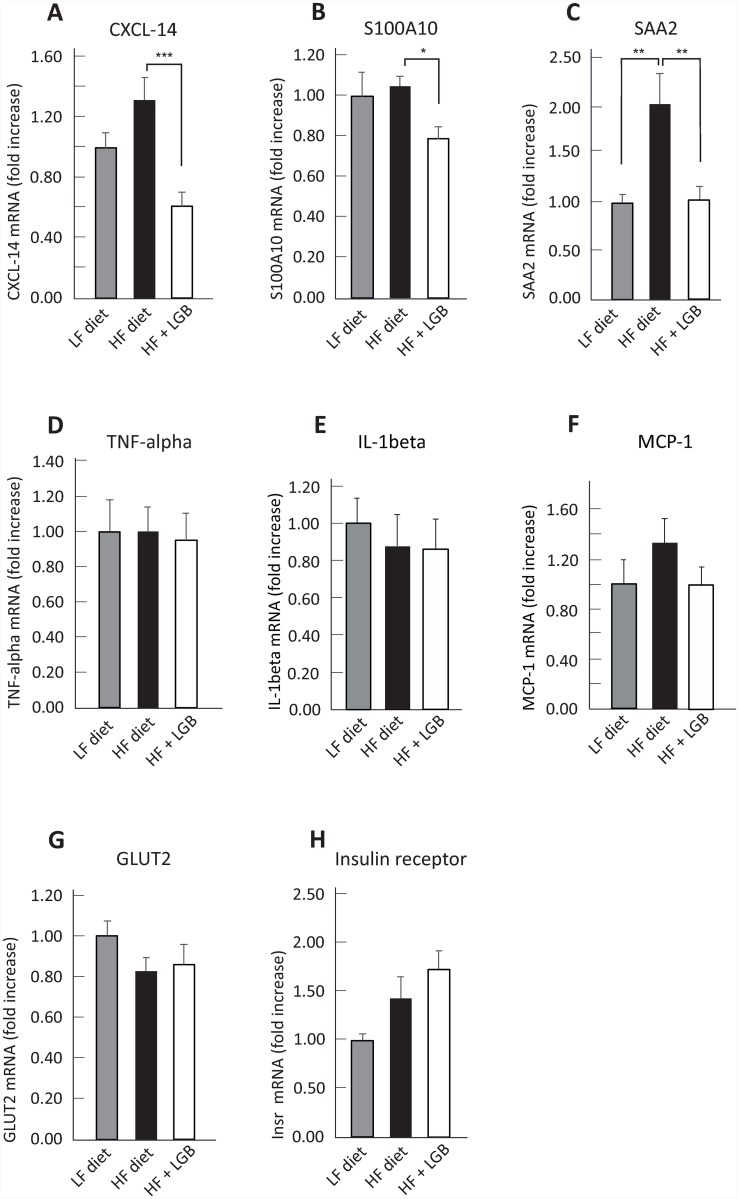
The expression of C-X-C motif chemokine ligand 14 (CXCL-14, A), S100 calcium-binding protein A10 (S100A10, B), serum amyloid A2 (SAA2, C), tumor necrosis alpha (TNF-α, D), interleukin 1β (IL-1β, E), monocyte chemoattractant protein 1 (MCP-1, F), glucose transporter 2 (GLUT2, G), and insulin receptor (H) in the liver. Animals received low-fat diet (LF diet, 10% energy from fat, grey columns), high-fat diet (HF diet, 46% energy from fat, black columns) or high-fat diet supplemented with lingonberry (HF + LGB diet, white columns). At the end of the study, liver samples were collected after 6 h fasting and the expression of the genes of interest were measured with RT-PCR. After normalizing to the housekeeping gene glyceraldehyde 3-phosphate dehydrogenase (GAPDH), the expression level in each sample was compared to the mean expression level in the LF-group, which was set as 1. One-way ANOVA with Bonferroni post-test was used in the statistical analysis. Differences between the groups are marked with *p < 0.05, **p < 0.01 and ***p < 0.001.

High-fat diet is known to induce metabolic and inflammatory changes in the liver. Therefore, we analyzed the expression of insulin receptor and glucose transporter GLUT2 as well as the inflammatory factors TNF-α, IL-1β, IL-6, MCP-1, SAA2, CXCL-14 and S100A10 in the liver by quantitative RT-PCR. As shown in the Fig 7, the hepatic expression of CXCL-14 (p < 0.001), S100A10 (p < 0.05), and SAA2 (p < 0.01) were significantly lower in the lingonberry supplemented high-fat diet group than in the high-fat control group, while IL-6 was under detection limit and no statistically significant differences in the expression of TNF-α, IL-1β, MCP-1, GLUT2 or insulin receptor were observed between the groups.

## Discussion

In the present study, we found that lingonberry supplementation prevented high-fat diet induced adverse effects on blood cholesterol, glucose and insulin levels as well as visceral fat gain in a murine model of obesity. In addition, the circulating levels of the pro-inflammatory adipocytokine leptin and the inflammatory acute phase reactant and biomarker serum amyloid A (SAA), as well as the alanine aminotransferase (ALT) activity were maintained at lower level by lingonberry supplementation, and the same was detected in hepatic expression of the inflammatory factors CXCL-14, S100A10 and SAA. To our knowledge, this is the first study in which such significant results have been obtained with an air-dried lingonberry powder. The results are remarkable also considering the rather short duration (6 weeks) of the study.

Obesity is a risk factor for glucose intolerance leading to diabetes, which is first detected as increased circulating insulin concentrations followed by increased fasting blood glucose levels [33,34]. In the present study, increased insulin and glucose levels were found already after six weeks on high-fat diet. Lingonberry supplementation prevented these effects. These findings are supported by previous studies with freeze-dried lingonberries and lingonberry extract on a longer follow-up [21–23,25,35] while bilberry supplementation did not have any effect on blood glucose levels [36]. Anthocyanins in the lingonberry powder could contribute to the beneficial effects on glucose tolerance as anthocyanin-rich supplements from Montmorency tart cherries have been reported to have insulin lowering effects in patients with metabolic syndrome [37] and purified blueberry anthocyanins reduced blood glucose levels in a murine model of obesity [38]. In the present study, lingonberry supplementation did not alter hepatic expression of the glucose transporter GLUT2 or insulin receptor. Further studies are needed to understand the mechanisms which mediate the positive effects of lingonberries on glucose tolerance in obesity.

Hyperlipidemia is another characteristic feature of obesity associated metabolic syndrome [4]. In the present study, lingonberry supplementation normalized the detrimental effects of the high-fat diet on the cholesterol levels, and triglycerides were also reduced. Proanthocyanidins present in lingonberries may explain, at least partly, the favorable effects on cholesterol levels [39–41]. In addition, the purified lingonberry anthocyanidin compound cyanidin-3-O-β-glucoside was found effective in decreasing total cholesterol levels in hypercholesterolemic ApoE deficient mice [42]. Lingonberries are also known to contain plant sterols at moderate levels of 20–30 mg/100 g of fresh weight [30,31] and they may contribute to the lipid lowering effects of lingonberries [43]. The lipid lowering effect seems to be characteristic for lingonberry among berries since freeze-dried blueberry and black raspberry [44] as well blackcurrant [21] were reported to enhance serum lipids in high-fat diet fed mice, while our findings are supported by previous studies with lingonberry containing diets [21,35,45]. High-fat treatment normally results in increased circulating triglycerides in humans [46], but in mice their levels may stay unchanged or decrease [21,38,45,47–49], and the former was found also in the present study. These effects on triglycerides may result from increased clearance of triglycerides and/or reduced production of triglycerides in the liver [47–49]. Increased ALT activity in the serum, as was also shown in the current study, supports the former assumption likewise associated with the development of nonalcoholic fatty liver disorder [50].

Adipokines are cytokine-like hormones originally found to be produced by adipose tissue and to regulate energy metabolism and appetite [51,52]. Leptin is a prototype adipokine, the concentrations of which are closely related to the amount of adipose tissue and body mass index [53,54]. This is the first study showing reduced leptin levels during lingonberry supplementation of high-fat diet but the results are supported by studies using blueberry anthocyanins [38,44]. It is not clear if this effect is simply a consequence of the moderate weight gain restraining effect of lingonberry supplementation or whether it has mechanistic relevancy. Anyhow, reduced leptin level may be considered as a beneficial effect because leptin has pro-inflammatory effects and contributes to the development of co-morbidities of obesity [55]. In addition, an increasing trend in adiponectin levels was seen in mice on lingonberry supplemented high-fat diet. Since adiponectin has insulin sensitizing effects [4], this is a positive signal and motivates further studies.

Obesity is associated with low-grade inflammation, which contributes to the obesity-related metabolic diseases. Serum amyloid A (SAA) is an example of the biomarkers and effectors of obesity-related inflammation, and it is involved in the mechanisms of lipid metabolism, atherosclerosis and acute phase response besides inflammation [56,57]. In the present study, circulating SAA concentrations, as well as its expression in the liver, were substantially increased during high-fat diet while lingonberry supplementation prevented these changes in a statistically significant manner. In addition, lingonberry supplementation reduced the hepatic expression of two other pro-inflammatory factors, i.e. the chemokine CXCL-14 and the S100 calcium-binding protein A10. These data indicate that lingonberry supplementation indeed suppresses the obesity-related inflammatory response.

Based on the present data, it remains unclear, which factor(s) present in the lingonberry powder may have caused the beneficial effects found in the current study, and further studies are needed to reveal the active constituents. The effects of lingonberry supplementation on the weight gain were moderate while it significantly prevented many of the metabolic and inflammatory adverse effects induced by the high-fat diet. Therefore, it is possible that lingonberry supplementation in the high-fat containing diet has anti-inflammatory consequences and shifts endogenous responses towards metabolically healthier obesity. Lingonberries contain several polyphenolic compounds, which have anti-inflammatory potential such as anthocyanins, ellagitannins, flavonols, phenolic acids and proanthocyanidins [58], flavonols and proanthocyanidins as well as anthocyanins forming the largest groups among the lingonberry polyphenols [58–62].

*Quercetin* is abundantly present in lingonberries [63]. Quercetin has been reported to have anti-inflammatory effects in *in vitro* and *in vivo* models [64–66]. Moreover, quercetin has also been suggested to have anti-adiposity activity *in vitro* by activating the AMPK signal pathway in adipocytes [67] and *in vivo* by preventing high-fat diet induced obesity in mice [68]. Lingonberries are also a rich source of *proanthocyanidins* [60,69] and *anthocyanins*, particularly cyanidin-3-galactoside, cyanidin-3-arabinoside, and cyanidin-3-glucoside [70,71]. Proanthocyanidins have been reported to contribute to the reduced weight gain and hypolipidemic effects of berry-derived preparations in animal models of obesity [40,72,73] while purified anthocyanins from mulberry (*M*. *australis* P.) and cherry (*Prunus avium* L.*)* prevented obesogenic diet induced weight gain, oxidative stress and inflammation in mice [74,75]. Lingonberry contains also the stilbenoid compound *resveratrol* (together with its glycosylated derivative piceid), which is a strong antioxidant with anti-inflammatory properties [76–78]. We have recently shown that resveratrol inhibits the production of nitric oxide and pro-inflammatory cytokines in activated macrophages and suppresses inflammatory responses *in vivo* [79]. Resveratrol has also been beneficial in animal models of obesity [80–82], and in a clinical study resveratrol supplementation improved glycemic control in patients with type 2 diabetes [83].

In conclusion, the present results show that lingonberry supplementation significantly prevents high-fat diet induced metabolic and inflammatory changes in a murine model of obesity. Further studies are needed to reveal the detailed molecular mechanisms and the active constituents responsible for the observed beneficial effects. The results encourage evaluation of lingonberries as a part of healthy diet against obesity and its comorbidities.
